# (*Z*)-4-[3-(3-Oxoquinuclidin-2-ylidene­meth­yl)-1*H*-indol-1-ylmeth­yl]benzo­nitrile

**DOI:** 10.1107/S1600536808030006

**Published:** 2008-10-04

**Authors:** Thirupathi Reddy Yerram Reddy, Narsimha Reddy Penthala, Sean Parkin, Peter A. Crooks

**Affiliations:** a Department of Pharmaceutical Sciences, College of Pharmacy, University of Kentucky, Lexington, KY 40536, USA; b Department of Chemistry, University of Kentucky, Lexington, KY 40506, USA

## Abstract

The title compound, C_24_H_21_N_3_O, was prepared by the reaction of (*Z*)-2-(1*H*-indol-3-ylmethyl­ene)-1-aza­bicyclo­[2.2.2]octan-3-one with α-bromo-*p*-toluonitrile, under phase-transfer catalytic (PTC) conditions using triethyl­benzyl­ammonium chloride and 50% *w*/*v* aqueous NaOH solution in dichloro­methane. The crystal structure indicates the presence of a double bond with *Z* geometry connecting the aza­bicyclic and indole groups.

## Related literature

For related structures, see: Mason *et al.* (2003 ▶); Zarza *et al.* (1988 ▶). For related bond angles, see: Wilson (1992 ▶). For related literature, see: Sekhar *et al.* (2003 ▶); Sonar *et al.* (2007 ▶).
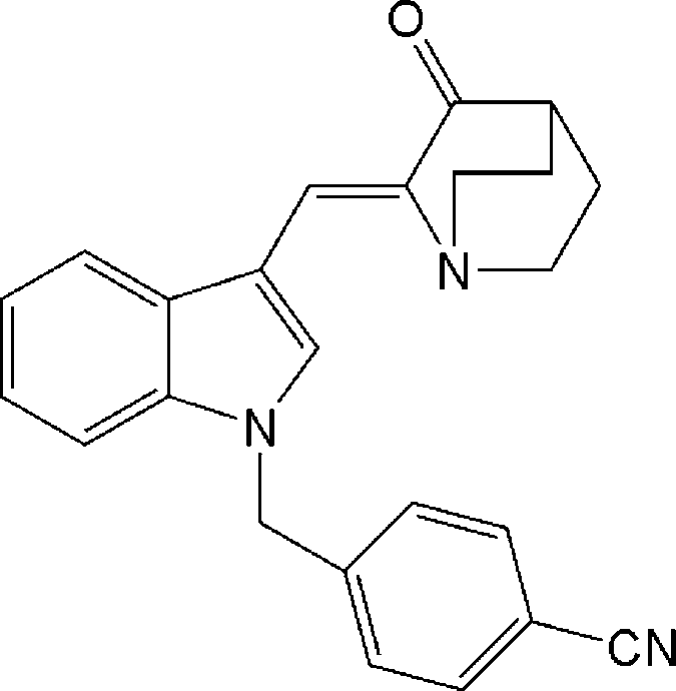


## Experimental

### 

#### Crystal data

C_24_H_21_N_3_O
*M* *_r_* = 367.44
Triclinic, 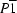

*a* = 9.0627 (2) Å
*b* = 10.7959 (3) Å
*c* = 11.6969 (4) Å
α = 99.1571 (13)°
β = 106.0935 (14)°
γ = 113.7908 (14)°
*V* = 957.16 (5) Å^3^
*Z* = 2
Mo *K*α radiation
μ = 0.08 mm^−1^
*T* = 90.0 (2) K
0.44 × 0.40 × 0.25 mm

#### Data collection

Nonius KappaCCD diffractometer
Absorption correction: multi-scan (*SCALEPACK*; Otwinowski & Minor, 1997 ▶) *T*_min_ = 0.966, *T*_max_ = 0.980
20970 measured reflections
4385 independent reflections
2935 reflections with *I* > 2σ(*I*)
*R*_int_ = 0.054

#### Refinement

*R*[*F* ^2^ > 2σ(*F* ^2^)] = 0.048
*wR*(*F* ^2^) = 0.128
*S* = 1.04
4385 reflections
253 parameters
H-atom parameters constrained
Δρ_max_ = 0.22 e Å^−3^
Δρ_min_ = −0.21 e Å^−3^

### 

Data collection: *COLLECT* (Nonius, 1998 ▶); cell refinement: *SCALEPACK* (Otwinowski & Minor, 1997 ▶); data reduction: *DENZO-SMN* (Otwinowski & Minor, 1997 ▶); program(s) used to solve structure: *SHELXS97* (Sheldrick, 2008 ▶); program(s) used to refine structure: *SHELXL97* (Sheldrick, 2008 ▶); molecular graphics: *XP* in *SHELXTL* (Sheldrick, 2008 ▶); software used to prepare material for publication: *SHELX97* and local procedures.

## Supplementary Material

Crystal structure: contains datablocks global, I. DOI: 10.1107/S1600536808030006/om2261sup1.cif

Structure factors: contains datablocks I. DOI: 10.1107/S1600536808030006/om2261Isup2.hkl

Additional supplementary materials:  crystallographic information; 3D view; checkCIF report

## Figures and Tables

**Fig. 1.**
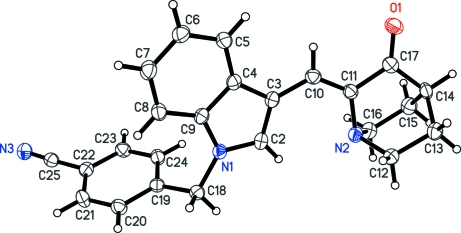
A view of the title molecule with the atom numbering scheme. Displacement ellipsoids are drawn at the 50% probability level.

**Fig. 2.**
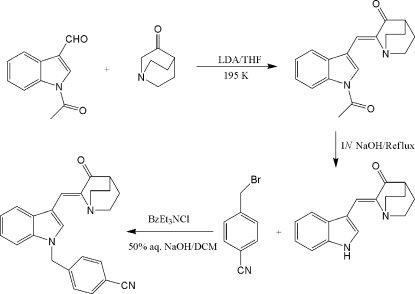
The formation of the title compound.

## supplementary crystallographic information

### Comment

The molecular structure and the atom-numbering scheme are illustrated in Fig. 1. The indole ring is planar with bond distances and angles comparable with those previously reported for other indole derivatives (Mason *et al.*, 2003; Zarza *et al.*, 1988). The compound is the *Z* isomer, with the C11—C17 bond in a *trans* position with respect to the C3—C10 bond. The olefinic bond (C10=C11) has a nearly planar atomic arrangement, since the r.m.s. deviation from the best plane passing through atoms N2, C11,C17, C10 and C3 is 0.0188 (9) Å. Deviations from ideal geometry are observed in the bond angles around atoms C3, C10 and C11. The C10=C11—C17 bond angle is close to the standard planar triangular value of 120°, whereas the C2=C3—C10, C3—C10=C11 and C10=C11—C17 bond angles are more distorted due to the strain induced by the C10=C11—C18=O1 conjugated double bond linkage. These bond angle deformations, which require little energy, are needed to relieve the strain of intramolecular interactions between non-bonded atoms. The azabicyclic system presents very small distortions around atoms N2, C13, C14, C15, C16 and C17. The value of the C2—C3—C10—C11 torsion angle [-5.1 (3)°] indicates the deviation of the indole ring from the plane of the double bond connected to the azabicyclic ring. The C3—C10 bond length, when compared with the standard value for a single bond connecting a C_ar_ atom to a C*sp*^2^ atom (1.470 (15) Å; Wilson, 1992), suggests extensive conjugation, beginning at atom O1 and extending through to the indole ring. The bond angles in the azabicyclic system at C13, C14 and C15 are, on average, smaller than the standard tetrahedral value of 109.5°, while the bond angles at C12 and C16 are, on average, slightly larger than the ideal tetrahedral bond angle.

There are no significant intermolecular hydrogen-bonding interactions in the packing of this compound. The packing is essentially stabilized *via* van der Waals forces.

### Experimental

To a stirred solution of diisopropylamine (1.923 g, 19 mmol) in THF (20 ml) at 273 K under nitrogen was added a solution of 2.0 *M* n-butyllithium (9 ml, 18.8 mmol) and the mixture stirred at 273 K for 30 min. To this solution at 273 K, was added 1-aza-bicyclo[2.2.2]octan-3-one hydrochloride (1.5 g, 9.28 mmol) in one portion and stirring continued until the mixture completely dissolved (20 min). The temperature was lowered to 195 K and a solution of 1-acetyl-1*H*-indole-3-carboxaldehyde (1.722 g, 9.2 mmol) in THF (25 ml) was added dropwise. Stirring was continued for 30 min at this temperature and then for 90 min at 0°C. The reaction mixture was poured into saturated NaHCO_3_ at 273 K and the resulting solution was extracted with CHCl_3_ (3 *x* 15 ml). The combined organic extracts were dried over anhydrous Na_2_SO_4_ and evaporated to afford (*Z*)-2-(1-acetyl- 1*H*-indol-3-ylmethylene)-1-azabicyclo[2.2.2]octan-3-one, which was subsequently refluxed with sodium hydroxide solution (25 ml, 1 N) for 30 min. The reaction mixture was cooled to room temperature, and the yellow solid that separated was collected by filtration, washed with cold water and dried to afford the (*Z*)-2-(1*H*-indol-3-ylmethylene) -1-azabicyclo[2.2.2]octan-3-one.

To a stirred mixture of (*Z*)-2-(1*H*-indol-3-ylmethylene) -1-azabicyclo[2.2.2]octan-3-one (1.0 g, 3.96 mmol), 50% *w*/*v* aqueous NaOH solution (1.52 g, 19 mmol) and benzyltriethylammonium chloride (0.172 g, 0.75 mmol) in dichloromethane (DCM, 25 ml) at room temperature was added α-bromo-*p*-tolunitrile (0.78 g, 4.0 mmol) in one portion, then the reaction mixture was stirred vigorously for 1 hr until no (*Z*)-2-(1*H*-indol-3-ylmethylene)-1-azabicyclo[2.2.2] octan-3-one was detected by TLC. The organic layer was separated, washed exhaustively with water, dried with Na_2_SO_4_ and evaporated to afford the crude product. Crystallization from methanol gave a yellow crystalline product of compound (I) that was suitable for X-ray analysis. ^1^H NMR (CDCl_3_): δ 1.98–2.04 (*m*, 4H), 2.60 (*p*, 1H), 2.92–2.99 (*m*, 2H), 3.08–3.18 (*m*, 2H), 5.43 (*s*, 2H), 7.10–7.23 (*m*, 5H), 7.45 (*s*, 1H), 7.57 (*d*, *J* = 7.2 Hz, 2H), 7.87 (*d*, *J* = 7.2 Hz, 1H), 8.37 (*s*, 1H); ^13^C NMR (CDCl_3_): δ 27.1, 41.1, 48.2, 50.8, 110.4, 111.5, 112.3, 118.0, 118.9, 119.9, 121.8, 123.5, 127.6, 129.3, 133.2, 134.2, 136.3, 141.7, 142.5, 205.7.

### Refinement

H atoms were found in difference Fourier maps and subsequently placed in idealized positions with constrained C—H distances of 0.99 Å (*R*_2_CH_2_), 0.99 Å (*R*_3_CH) and 0.95 Å (C_Ar_H) with *U*_iso_(H) values set to 1.2*U*_eq_ of the attached C atom.

### Crystal data

**Table:**

C_24_H_21_N_3_O	*Z* = 2
*M**_r_* = 367.44	*F*(000) = 388
Triclinic, *P*1	*D*_x_ = 1.275 Mg m^−^^3^
Hall symbol: -P 1	Mo *K*α radiation, λ = 0.71073 Å
*a* = 9.0627 (2) Å	Cell parameters from 4341 reflections
*b* = 10.7959 (3) Å	θ = 1.0–27.5°
*c* = 11.6969 (4) Å	µ = 0.08 mm^−^^1^
α = 99.1571 (13)°	*T* = 90 K
β = 106.0935 (14)°	Irregular block, colourless
γ = 113.7908 (14)°	0.44 × 0.40 × 0.25 mm
*V* = 957.16 (5) Å^3^	

### Data collection

**Table:**

Nonius KappaCCD diffractometer	4385 independent reflections
Radiation source: fine-focus sealed tube	2935 reflections with *I* > 2σ(*I*)
graphite	*R*_int_ = 0.054
Detector resolution: 18 pixels mm^-1^	θ_max_ = 27.5°, θ_min_ = 1.9°
ω scans at fixed χ = 55°	*h* = −11→11
Absorption correction: multi-scan (SCALEPACK; Otwinowski & Minor, 1997)	*k* = −14→13
*T*_min_ = 0.966, *T*_max_ = 0.980	*l* = 0→15
20970 measured reflections	

### Refinement

**Table:**

Refinement on *F*^2^	Primary atom site location: structure-invariant direct methods
Least-squares matrix: full	Secondary atom site location: difference Fourier map
*R*[*F*^2^ > 2σ(*F*^2^)] = 0.048	Hydrogen site location: inferred from neighbouring sites
*wR*(*F*^2^) = 0.128	H-atom parameters constrained
*S* = 1.04	*w* = 1/[σ^2^(*F*_o_^2^) + (0.0661*P*)^2^ + 0.0538*P*] where *P* = (*F*_o_^2^ + 2*F*_c_^2^)/3
4385 reflections	(Δ/σ)_max_ = 0.004
253 parameters	Δρ_max_ = 0.22 e Å^−^^3^
0 restraints	Δρ_min_ = −0.21 e Å^−^^3^

### Special details

**Table:**

Geometry. All e.s.d.'s (except the e.s.d. in the dihedral angle between two l.s. planes) are estimated using the full covariance matrix. The cell e.s.d.'s are taken into account individually in the estimation of e.s.d.'s in distances, angles and torsion angles; correlations between e.s.d.'s in cell parameters are only used when they are defined by crystal symmetry. An approximate (isotropic) treatment of cell e.s.d.'s is used for estimating e.s.d.'s involving l.s. planes.
Refinement. Refinement of *F*^2^ against ALL reflections. The weighted *R*-factor *wR* and goodness of fit *S* are based on *F*^2^, conventional *R*-factors *R* are based on *F*, with *F* set to zero for negative *F*^2^. The threshold expression of *F*^2^ > 2σ(*F*^2^) is used only for calculating *R*-factors(gt) *etc*. and is not relevant to the choice of reflections for refinement. *R*-factors based on *F*^2^ are statistically about twice as large as those based on *F*, and *R*- factors based on ALL data will be even larger.

### Fractional atomic coordinates and isotropic or equivalent isotropic displacement parameters (Å^2^)

**Table:**

	*x*	*y*	*z*	*U*_iso_*/*U*_eq_	
N1	0.63840 (16)	0.43150 (13)	0.54539 (11)	0.0220 (3)	
N2	0.29373 (16)	0.01430 (13)	0.25488 (12)	0.0241 (3)	
N3	1.56395 (18)	0.85155 (14)	1.00029 (13)	0.0306 (3)	
O1	0.00836 (14)	−0.22274 (12)	0.37598 (11)	0.0320 (3)	
C2	0.53780 (19)	0.29481 (16)	0.46742 (15)	0.0243 (4)	
H2	0.5404	0.2614	0.3882	0.029*	
C3	0.43160 (19)	0.21170 (16)	0.51985 (14)	0.0228 (3)	
C4	0.47183 (19)	0.30524 (16)	0.63990 (14)	0.0215 (3)	
C5	0.4079 (2)	0.28731 (16)	0.73508 (15)	0.0251 (4)	
H5	0.3221	0.1967	0.7294	0.030*	
C6	0.4719 (2)	0.40387 (17)	0.83761 (15)	0.0287 (4)	
H6	0.4307	0.3925	0.9036	0.034*	
C7	0.5965 (2)	0.53862 (17)	0.84652 (16)	0.0296 (4)	
H7	0.6366	0.6171	0.9177	0.036*	
C8	0.6620 (2)	0.55952 (16)	0.75394 (15)	0.0258 (4)	
H8	0.7465	0.6508	0.7598	0.031*	
C9	0.59912 (19)	0.44119 (16)	0.65166 (14)	0.0221 (3)	
C10	0.29595 (19)	0.06574 (15)	0.46778 (15)	0.0237 (4)	
H10	0.2459	0.0273	0.5236	0.028*	
C11	0.23045 (19)	−0.02341 (15)	0.35161 (14)	0.0225 (3)	
C12	0.1480 (2)	0.00383 (16)	0.15034 (15)	0.0266 (4)	
H12A	0.1881	0.0271	0.0822	0.032*	
H12B	0.1124	0.0744	0.1797	0.032*	
C13	−0.0109 (2)	−0.14677 (17)	0.09871 (16)	0.0301 (4)	
H13A	−0.1140	−0.1411	0.1069	0.036*	
H13B	−0.0382	−0.1885	0.0090	0.036*	
C14	0.03321 (19)	−0.24051 (16)	0.17351 (15)	0.0259 (4)	
H14	−0.0676	−0.3383	0.1441	0.031*	
C15	0.1944 (2)	−0.24547 (17)	0.15857 (16)	0.0303 (4)	
H15A	0.1687	−0.2881	0.0694	0.036*	
H15B	0.2270	−0.3043	0.2066	0.036*	
C16	0.3455 (2)	−0.09142 (16)	0.20803 (15)	0.0280 (4)	
H16A	0.4442	−0.0848	0.2765	0.034*	
H16B	0.3857	−0.0684	0.1399	0.034*	
C17	0.0820 (2)	−0.16865 (16)	0.30955 (15)	0.0249 (4)	
C18	0.76263 (19)	0.55154 (16)	0.52187 (14)	0.0235 (3)	
H18A	0.7227	0.6243	0.5193	0.028*	
H18B	0.7662	0.5186	0.4392	0.028*	
C19	0.94360 (19)	0.61802 (15)	0.62085 (14)	0.0222 (3)	
C20	1.04167 (19)	0.76546 (16)	0.67051 (15)	0.0264 (4)	
H20	0.9969	0.8237	0.6379	0.032*	
C21	1.2034 (2)	0.82836 (17)	0.76673 (15)	0.0284 (4)	
H21	1.2684	0.9290	0.8012	0.034*	
C22	1.26979 (19)	0.74218 (16)	0.81242 (14)	0.0238 (4)	
C23	1.1756 (2)	0.59485 (16)	0.76076 (15)	0.0257 (4)	
H23	1.2224	0.5365	0.7905	0.031*	
C24	1.0130 (2)	0.53398 (16)	0.66567 (15)	0.0256 (4)	
H24	0.9482	0.4334	0.6307	0.031*	
C25	1.4347 (2)	0.80457 (16)	0.91607 (15)	0.0251 (4)	

### Atomic displacement parameters (Å^2^)

**Table:**

	*U*^11^	*U*^22^	*U*^33^	*U*^12^	*U*^13^	*U*^23^
N1	0.0188 (6)	0.0228 (7)	0.0201 (7)	0.0072 (6)	0.0063 (5)	0.0052 (6)
N2	0.0228 (7)	0.0244 (7)	0.0227 (7)	0.0105 (6)	0.0066 (6)	0.0063 (6)
N3	0.0275 (8)	0.0302 (8)	0.0286 (8)	0.0114 (6)	0.0075 (7)	0.0067 (6)
O1	0.0327 (7)	0.0271 (6)	0.0347 (7)	0.0098 (5)	0.0162 (6)	0.0113 (5)
C2	0.0227 (8)	0.0239 (8)	0.0208 (8)	0.0099 (7)	0.0046 (7)	0.0026 (7)
C3	0.0214 (8)	0.0241 (8)	0.0208 (8)	0.0111 (7)	0.0055 (7)	0.0052 (7)
C4	0.0190 (8)	0.0230 (8)	0.0211 (8)	0.0108 (7)	0.0043 (6)	0.0068 (7)
C5	0.0222 (8)	0.0252 (9)	0.0251 (9)	0.0091 (7)	0.0081 (7)	0.0079 (7)
C6	0.0281 (9)	0.0324 (9)	0.0242 (9)	0.0123 (8)	0.0119 (7)	0.0069 (7)
C7	0.0298 (9)	0.0288 (9)	0.0255 (9)	0.0116 (8)	0.0106 (8)	0.0022 (7)
C8	0.0231 (8)	0.0230 (8)	0.0249 (9)	0.0081 (7)	0.0060 (7)	0.0046 (7)
C9	0.0199 (8)	0.0248 (8)	0.0199 (8)	0.0108 (7)	0.0051 (6)	0.0059 (7)
C10	0.0245 (8)	0.0229 (8)	0.0250 (9)	0.0117 (7)	0.0092 (7)	0.0091 (7)
C11	0.0214 (8)	0.0207 (8)	0.0243 (9)	0.0094 (7)	0.0075 (7)	0.0075 (7)
C12	0.0262 (9)	0.0262 (9)	0.0245 (9)	0.0114 (7)	0.0067 (7)	0.0082 (7)
C13	0.0244 (9)	0.0304 (9)	0.0287 (9)	0.0106 (7)	0.0041 (7)	0.0092 (7)
C14	0.0210 (8)	0.0187 (8)	0.0273 (9)	0.0043 (7)	0.0036 (7)	0.0044 (7)
C15	0.0324 (9)	0.0269 (9)	0.0300 (10)	0.0148 (8)	0.0100 (8)	0.0060 (7)
C16	0.0260 (9)	0.0296 (9)	0.0286 (9)	0.0139 (7)	0.0101 (7)	0.0074 (7)
C17	0.0229 (8)	0.0238 (8)	0.0300 (9)	0.0129 (7)	0.0090 (7)	0.0105 (7)
C18	0.0207 (8)	0.0240 (8)	0.0230 (8)	0.0081 (7)	0.0078 (7)	0.0082 (7)
C19	0.0215 (8)	0.0232 (8)	0.0210 (8)	0.0083 (7)	0.0104 (7)	0.0068 (7)
C20	0.0243 (8)	0.0239 (9)	0.0293 (9)	0.0108 (7)	0.0077 (7)	0.0101 (7)
C21	0.0243 (9)	0.0198 (8)	0.0318 (10)	0.0060 (7)	0.0068 (7)	0.0040 (7)
C22	0.0208 (8)	0.0272 (9)	0.0222 (8)	0.0104 (7)	0.0084 (7)	0.0068 (7)
C23	0.0247 (9)	0.0252 (9)	0.0285 (9)	0.0128 (7)	0.0091 (7)	0.0105 (7)
C24	0.0242 (8)	0.0223 (8)	0.0256 (9)	0.0084 (7)	0.0081 (7)	0.0049 (7)
C25	0.0251 (9)	0.0241 (9)	0.0270 (9)	0.0109 (7)	0.0115 (8)	0.0090 (7)

### Geometric parameters (Å, °)

**Table:**

N1—C2	1.3659 (19)	C12—H12B	0.9900
N1—C9	1.3845 (19)	C13—C14	1.537 (2)
N1—C18	1.4597 (18)	C13—H13A	0.9900
N2—C11	1.4466 (19)	C13—H13B	0.9900
N2—C12	1.4804 (19)	C14—C17	1.507 (2)
N2—C16	1.4825 (19)	C14—C15	1.539 (2)
N3—C25	1.150 (2)	C14—H14	1.0000
O1—C17	1.2280 (18)	C15—C16	1.547 (2)
C2—C3	1.383 (2)	C15—H15A	0.9900
C2—H2	0.9500	C15—H15B	0.9900
C3—C10	1.443 (2)	C16—H16A	0.9900
C3—C4	1.446 (2)	C16—H16B	0.9900
C4—C5	1.396 (2)	C18—C19	1.510 (2)
C4—C9	1.409 (2)	C18—H18A	0.9900
C5—C6	1.380 (2)	C18—H18B	0.9900
C5—H5	0.9500	C19—C24	1.387 (2)
C6—C7	1.401 (2)	C19—C20	1.392 (2)
C6—H6	0.9500	C20—C21	1.384 (2)
C7—C8	1.379 (2)	C20—H20	0.9500
C7—H7	0.9500	C21—C22	1.395 (2)
C8—C9	1.391 (2)	C21—H21	0.9500
C8—H8	0.9500	C22—C23	1.392 (2)
C10—C11	1.342 (2)	C22—C25	1.442 (2)
C10—H10	0.9500	C23—C24	1.384 (2)
C11—C17	1.482 (2)	C23—H23	0.9500
C12—C13	1.549 (2)	C24—H24	0.9500
C12—H12A	0.9900		
			
C2—N1—C9	108.68 (12)	H13A—C13—H13B	108.3
C2—N1—C18	127.10 (13)	C17—C14—C13	107.70 (13)
C9—N1—C18	124.18 (13)	C17—C14—C15	107.79 (13)
C11—N2—C12	108.02 (12)	C13—C14—C15	108.08 (13)
C11—N2—C16	108.35 (11)	C17—C14—H14	111.0
C12—N2—C16	108.05 (12)	C13—C14—H14	111.0
N1—C2—C3	110.60 (14)	C15—C14—H14	111.0
N1—C2—H2	124.7	C14—C15—C16	108.19 (12)
C3—C2—H2	124.7	C14—C15—H15A	110.1
C2—C3—C10	129.90 (14)	C16—C15—H15A	110.1
C2—C3—C4	105.74 (13)	C14—C15—H15B	110.1
C10—C3—C4	124.17 (13)	C16—C15—H15B	110.1
C5—C4—C9	119.08 (14)	H15A—C15—H15B	108.4
C5—C4—C3	133.84 (14)	N2—C16—C15	112.41 (12)
C9—C4—C3	107.03 (13)	N2—C16—H16A	109.1
C6—C5—C4	118.62 (15)	C15—C16—H16A	109.1
C6—C5—H5	120.7	N2—C16—H16B	109.1
C4—C5—H5	120.7	C15—C16—H16B	109.1
C5—C6—C7	121.38 (15)	H16A—C16—H16B	107.9
C5—C6—H6	119.3	O1—C17—C11	124.83 (15)
C7—C6—H6	119.3	O1—C17—C14	124.67 (14)
C8—C7—C6	121.27 (15)	C11—C17—C14	110.51 (13)
C8—C7—H7	119.4	N1—C18—C19	112.15 (12)
C6—C7—H7	119.4	N1—C18—H18A	109.2
C7—C8—C9	117.14 (15)	C19—C18—H18A	109.2
C7—C8—H8	121.4	N1—C18—H18B	109.2
C9—C8—H8	121.4	C19—C18—H18B	109.2
N1—C9—C8	129.54 (14)	H18A—C18—H18B	107.9
N1—C9—C4	107.93 (13)	C24—C19—C20	119.08 (14)
C8—C9—C4	122.50 (15)	C24—C19—C18	120.83 (14)
C11—C10—C3	129.13 (15)	C20—C19—C18	120.07 (13)
C11—C10—H10	115.4	C21—C20—C19	120.97 (14)
C3—C10—H10	115.4	C21—C20—H20	119.5
C10—C11—N2	123.45 (14)	C19—C20—H20	119.5
C10—C11—C17	122.77 (14)	C20—C21—C22	119.17 (15)
N2—C11—C17	113.76 (13)	C20—C21—H21	120.4
N2—C12—C13	111.92 (12)	C22—C21—H21	120.4
N2—C12—H12A	109.2	C23—C22—C21	120.39 (14)
C13—C12—H12A	109.2	C23—C22—C25	119.24 (14)
N2—C12—H12B	109.2	C21—C22—C25	120.35 (14)
C13—C12—H12B	109.2	C24—C23—C22	119.51 (14)
H12A—C12—H12B	107.9	C24—C23—H23	120.2
C14—C13—C12	108.64 (13)	C22—C23—H23	120.2
C14—C13—H13A	110.0	C23—C24—C19	120.82 (14)
C12—C13—H13A	110.0	C23—C24—H24	119.6
C14—C13—H13B	110.0	C19—C24—H24	119.6
C12—C13—H13B	110.0	N3—C25—C22	178.06 (17)
			
C9—N1—C2—C3	−0.32 (16)	C16—N2—C12—C13	59.45 (16)
C18—N1—C2—C3	−177.95 (13)	N2—C12—C13—C14	−0.11 (18)
N1—C2—C3—C10	174.58 (14)	C12—C13—C14—C17	56.92 (16)
N1—C2—C3—C4	−0.44 (16)	C12—C13—C14—C15	−59.29 (17)
C2—C3—C4—C5	178.37 (16)	C17—C14—C15—C16	−57.07 (16)
C10—C3—C4—C5	3.0 (3)	C13—C14—C15—C16	59.08 (16)
C2—C3—C4—C9	1.02 (16)	C11—N2—C16—C15	57.11 (16)
C10—C3—C4—C9	−174.37 (14)	C12—N2—C16—C15	−59.69 (16)
C9—C4—C5—C6	0.0 (2)	C14—C15—C16—N2	0.19 (18)
C3—C4—C5—C6	−177.08 (15)	C10—C11—C17—O1	−2.1 (2)
C4—C5—C6—C7	1.0 (2)	N2—C11—C17—O1	179.80 (13)
C5—C6—C7—C8	−1.0 (2)	C10—C11—C17—C14	177.55 (14)
C6—C7—C8—C9	0.0 (2)	N2—C11—C17—C14	−0.58 (17)
C2—N1—C9—C8	−176.79 (15)	C13—C14—C17—O1	121.88 (16)
C18—N1—C9—C8	0.9 (2)	C15—C14—C17—O1	−121.72 (16)
C2—N1—C9—C4	0.97 (16)	C13—C14—C17—C11	−57.74 (16)
C18—N1—C9—C4	178.69 (12)	C15—C14—C17—C11	58.66 (16)
C7—C8—C9—N1	178.54 (15)	C2—N1—C18—C19	−122.47 (16)
C7—C8—C9—C4	1.1 (2)	C9—N1—C18—C19	60.24 (18)
C5—C4—C9—N1	−179.04 (12)	N1—C18—C19—C24	42.68 (19)
C3—C4—C9—N1	−1.23 (16)	N1—C18—C19—C20	−135.82 (14)
C5—C4—C9—C8	−1.1 (2)	C24—C19—C20—C21	−2.6 (2)
C3—C4—C9—C8	176.73 (14)	C18—C19—C20—C21	175.96 (14)
C2—C3—C10—C11	−5.1 (3)	C19—C20—C21—C22	1.3 (2)
C4—C3—C10—C11	169.08 (15)	C20—C21—C22—C23	0.9 (2)
C3—C10—C11—N2	2.1 (2)	C20—C21—C22—C25	−177.41 (14)
C3—C10—C11—C17	−175.87 (14)	C21—C22—C23—C24	−1.7 (2)
C12—N2—C11—C10	−119.06 (15)	C25—C22—C23—C24	176.58 (14)
C16—N2—C11—C10	124.13 (15)	C22—C23—C24—C19	0.4 (2)
C12—N2—C11—C17	59.06 (15)	C20—C19—C24—C23	1.7 (2)
C16—N2—C11—C17	−57.76 (16)	C18—C19—C24—C23	−176.82 (14)
C11—N2—C12—C13	−57.57 (16)		
